# Prevalence, habits and personal attitudes towards smoking among health care professionals

**DOI:** 10.1186/s12995-017-0166-5

**Published:** 2017-07-26

**Authors:** Brankica Juranić, Željko Rakošec, Jelena Jakab, Štefica Mikšić, Suzana Vuletić, Marul Ivandić, Ivka Blažević

**Affiliations:** 1 Faculty of Medicine, Josip Juraj Strossmayer University of Osijek, Cara Hadrijana 10/E, HR-31000 Osijek, Croatia; 2 Department of Culturology, Josip Juraj Strossmayer University of Osijek, Trg Svetog Trojstva 3, Osijek, Croatia; 3 Catholic Faculty of Theology in Đakovo, Josip Juraj Strossmayer University of Osijek, Petra Preradovića 17, Đakovo, Croatia; 4 Public Health Centre Osijek, Park kralja Petra Krešimira IV. 6, HR-31000 Osijek, Croatia

**Keywords:** Attitude, Habits, Prevalence, Smoking

## Abstract

**Background:**

Tobacco use is the second major cause of morbidity and the 4th most common health risk factor in the world. Medical professionals have a critical role in the process of smoking cessation both as advisers and behavioural models for the citizens. The aim of this study was to investigate the prevalence of smoking among health care professionals, their smoking habits and personal attitudes toward smoking, role and the responsibility of health care professionals in the prevention of smoking.

**Results:**

Out of the total number of examinees, 175 (35,1%) are active smokers, 29 (5,8%) are former smokers, and 295 (59,1%) are non-smokers. Nurses with secondary education disagree the most with the claim that passive smoking is more harmful to health (χ2 test, *p* = .008), also with the claim that the introduced Smoking Act is fair to smokers (χ2 test, *p* = .021). More nurses with secondary education disagree completely or partially that one should pay attention to smoking in the presence of non-smokers (χ2 test, *p* = .012).

**Conclusion:**

Training programs for health care workers are needed to improve their ability in smoking cessation techniques to provide active support to their patients.

## Background

Worldwide, tobacco use is the second major cause of morbidity and the 4th most common health risk factor in the world. It causes diseases such as lung cancer, chronic obstructive pulmonary disease and coronary heart disease [1, 2].

All modern states are responsible for the preservation and improvement of their citizen’s health and are therefore obliged to undertake systematic legal action to fight tobacco smoking. A comprehensive approach to reduction of this global pandemic is a priority, which can be seen in EU anti-smoking strategy [3]. This strategy offers extensive smoking prevention and cessation activities, including education measures and restrictions on tobacco marketing [4–7].

According to “The Survey on Tobacco use in the adult population of Croatia” conducted in 2014/2015., 31% of Croatians are smokers. As an EU member country, Croatia implements a stricter enforcement of new laws against smoking which was first brought in 1999. The Act on the Restriction of the Use of Tobacco Products imposes a total smoking ban in all workplaces, all public areas where people work or stay, open areas such as school yards, hospital areas, sports stadiums, arenas, open-air theatres, tram and bus stations. Smoking is also banned in all enclosed public areas including bars, restaurants and cafes [8].

It is of major importance to assess habits and attitudes toward smoking of medical professionals, given their critical role in the process of smoking cessation both as advisers and behavioural models for the citizens [9]. Especially important is the way they see themselves in a role of helping smokers who wish to quit [10]. This mostly applies to nurses, given the fact that they represent the biggest working group among healthcare professionals, thus having a major impact on their colleagues and patients regarding smoking habits.

Two important findings emerged from a number of studies are that health professionals have more success in convincing patients to stop smoking if they are not smokers [11]; and smokers who rely on a support and advice of their healthcare provider have more chances to quit than those who try it on their own [12–14]. Despite all this, and despite the well-known damages of smoking, studies still show a high prevalence of smokers among health professionals [15–19], meaning they are not a good example for their patients [20, 21]. In some European countries, smoking prevalence among healthcare professionals is higher or at least equal to the prevalence in general population. For example, smoking prevalence among Italian physicians is 28%, this percentage among physicians in France is 34%, while there is 40% of Greek and Turkish physicians who smoke [22]. Also, studies have shown that nurses smoke more than physicians, especially the ones with the secondary level of education [23–28]. Bearing in mind that physicians and nurses are considered an example to community and colleagues [29], it is important that hospitals represent a place where the culture of health promotion and smoking cessation is developed [30].

The aim of this study was to investigate the prevalence of smoking among health care professionals, their smoking habit and personal attitudes toward smoking, in order to rise the responsibility of health care professionals in the prevention of smoking.

## Methods

### Study design

This study is designed as a descriptive cross-sectional survey.

### Setting and sample

The study was carried out in 11 clinics, 8 departments and 4 Clinical Institutes of the University Hospital Centre Osijek. The total number of the examinees is 499, what makes up 23% of the total number of the health care professionals employed at the University Hospital Centre in 2015. A total of 620 surveys were distributed and 499 (80.4%) responded.

### Ethical consideration

Prior to their participation in the research, examinees have been informed that the participation in the survey is voluntary and anonymous, also about what is expected from them and about the aim of the research, what has also been specified on the questionnaire. The approval from the University Clinical Hospital Centre Osijek’s Ethical Committee was acquired before the conduction of the study.

### Measurements/instruments

The study was conducted by means of an anonymous questionnaire which consists of general information, basic habits related to smoking, and personal attitudes related to smoking. The questionnaire was made on the basis of theoretical knowledge, literature review and prior experience. This questionnaire was not validated before this research.

The first part of the questionnaire contains 7 questions (sex, year of birth, occupation, years of work experience, respiratory tract difficulties). The second part of the questionnaire contains questions related to personal smoking habits – how long have you been smoking, how many cigarettes a day, do you smoke in your workplace, do you smoke in the presence of your patients. The third part of the questionnaire contains 14 questions related to personal attitudes toward smoking, at which point the examinees answered the questions using evaluation (from 1 to 5).

### Statistical analysis

Numerical data was described by measures of centre and spread. The normality of the distribution of the observed numeric variables was tested using the Kolmogorov–Smirnov test. The categorical variables are described by absolute and relative frequencies. The differences between the categorical dependent variables were tested by chi-square (χ 2) test and by Fisher’s exact test.

To evaluate the structure of the questionnaire about attitudes toward smoking we used the common factor analysis method with orthogonal rotation (Varimax). We have extracted two significant factors by Kaiser-Guttman and Cattell criterion. Those two factors explain the total of 53.99% variance, so we can conclude that the questionnaire has a valid structure (Table 1).

**Table 1:** Common factor models after orthogonal rotation

	Factor	Factor
	1	2
osp3	.715	
osp2	.687	
osp4	.647	
osp7	.632	
osp1	.582	
osp5	.565	
osp14		.954
osp13		.848
osp15		.649
Characteristic values	2.61	2.25
% of Variance	29.02	24.98
Cumulative %	53.99	

Extraction Method: Principal Axis Factoring

Rotation Method: Varimax with Kaiser Normalization

The level *p* < 0.05 was chosen for the statistical significance assessment of the obtained results. Statistics software package Statistica for Windows 2005 (version 7.1, StatSoft Inc., Tulsa, OK, USA) had been used).

## Results

There were 499 examinees included in the research, 413 (82.8%) of which females. The average age of the examinees is 39.8 (±11.54) years. According to the workplace, the most numerous are nurses and medical technicians with secondary education - 253 (50.7%), followed by 97 (19.4%) nurses/technicians with an associate degree, significantly more females than males (χ^2^ test, *p* < .001). Regarding years of service, 134 (26.9%) examinees had 11 to 20 years of service, and 73 (14.6%) examinees had more than 30 years of service.

The reliability of the questionnaire was verified by the Cronbach Alpha reliability coefficient. For the first factor the value is .82, and for the second is .88, suggesting both of them have relatively high internal consistency.

Out of the total number of examinees, there are 175 (35.1%) active smokers, 9 (5.8%) former smokers, and 295 (59.1%) non-smokers. There is no significant difference between wether someone is a smoker and which workplace they hold. The greatest number of smokers, 59 (11.8%), started smoking between 11 to 20 years ago, and 12 (2.4%) of the examinees smoke longer than 30 years. Concerning a number of cigarettes smoked, 86 (17.2%) examinees smoke 10 to 20 cigarettes per day while only 2 (0.4%) smoke over 30 cigarettes per day. In public areas smoke 99 (19.8%) examinees, significantly more females than males (Fisher’s exact test, *p* = .023); 134 (26.9%) examinees smoke in their workplace, while only 1 (0.2%) examinee smokes in the presence of the patients.

There are 11 (2.2%) examinees who disagree with the claim that passive smoking is bad for the health, significantly more nurses/technicians (chi-square, *p* = .008) (Table 2). In addition, significantly more nurses/ technicians with secondary education consider that the recently introduced Smoking Ban Act is not fair to the smokers (Table 3). More nurses do not agree fully or partially about wether one should pay attention to smoking in the presence of non-smokers (Table 4).

**Table 2:** Attitudes toward harmful effects of smoking

	Resident physicians	Specialist physicians	Trainee nurses	Nurses with secondary education	Nurses with associate degree	Total	p*
	N (%)	N (%)	N (%)	N (%)	N (%)	N (%)	
Smoking is harmful to health							
Strongly disagree	0 (0.0)	0 (0.0)	0 (0.0)	4 (1.6)	1 (1.0)	5 (1.0)	.004
Disagree	0 (0.0)	0 (0.0)	0 (0.0)	14 (5.5)	2 (2.1)	16 (3.2)	
Undecided	0 (0.0)	0 (0.0)	1 (2.6)	18 (7.1)	4 (4.1)	23 (4.6)	
Agree	0 (0.0)	5 (6.4)	8 (20.5)	35 (13.8)	11 (11.3)	59 (11.8)	
Strongly agree	32 (100)	73 (93.6)	30 (76.9)	182 (71.9)	79 (81.4)	396 (79.4)	
Second-hand smoking is harmful to health							
Strongly disagree	0 (0.0)	1 (1.3)	1 (2.6)	8 (3.2)	1 (1.0)	11 (2.2)	.008
Disagree	0 (0.0)	0 (0.0)	1 (2.6)	15 (5.9)	3 (3.1)	19 (3.8)	
Undecided	1 (3.1)	2 (2.6)	1 (2.6)	22 (8.8)	10 (10.3)	36 (7.2)	
Agree	4 (12.5)	12 (15.4)	10 (25.6)	63 (25.1)	14 (14.4)	103 (20.7)	
Strongly agree	27 (84.4)	63 (80.8)	26 (66.7)	143 (56.9)	69 (71.1)	328 (66.0)	

*chi-square

**Table 3:** Attitudes toward Smoking Ban Act

	Resident physicians	Spcialist physicians	Trainee nurses	Nurses with secondary education	Nurses with associate degree	Total	p*
	N (%)	N (%)	N (%)	N (%)	N (%)	N (%)	
Smoking Ban Act is fair to the non-smokers							
Strongly disagree	0 (0.0)	1 (1.3)	3 (7.7)	24 (9.6)	4 (4.2)	32 (6.5)	
Disagree	0 (0.0)	2 (2.6)	0 (0.0)	20 (8.0)	5 (5.2)	27 (5.5)	.021
Undecided	3 (9.4)	6 (7.8)	8 (20.5)	29 (11.6)	10 (10.4)	56 (11.3)	
Agree	108 (31.3)	14 (18.2)	5 (12.8)	51 (20.4)	21 (21.9)	101 (20.5)	
Strongly agree	19 (59.4)	54 (70.1)	23 (59.0)	126 (50.4)	56 (58.3)	278 (56.3)	
Smoking Ban Act is fair to the smokers							
Strongly disagree	3 (9.7)	6 (7.8)	5 (12.8)	52 (20.9)	19 (19.6)	85 (17.2)	.005
Disagree	3 (9.7)	9 (11.7)	3 (7.7)	43 (17.3)	9 (9.3)	67 (13.6)	
Undecided	7 (22.6)	9 (11.7)	13 (33.3)	45 (18.1)	19 (19.6)	93 (18.9)	
Agree	10 (32.3)	21 (27.3)	9 (23.1)	61 (24.5)	20 (20.6)	121 (24.5)	
Strongla agree	8 (25.8)	32 (41.6)	9 (23.1)	48 (19.3)	30 (30.9)	127 (25.8)	
Total	32 (100)	78 (100)	39 (100)	251(100)	97 (100)	497 (100)	

*chi-square

**Table 4:** Attitudes toward smoking in front of non-smokers and children

	Resident physicians	Specialist physicians	Trainee nurses	Nurses with secondary education	Nurses with associate degree	Total	P*
	N (%)	N (%)	N (%)	N (%)	N (%)	N (%)	
One should pay attention to smoking in presence of non-smokers							
Strongly disagree	0 (0.0)	1 (1.3)	2 (5.1)	15 (5.9)	3 (3.1)	21 (4.2)	.012
Disagree	0 (0.0)	1 (1.3)	0 (0.0)	12 (4.8)	4 (4.2)	17 (3.4)	
Undecided	2 (6.2)	1 (1.3)	4 (10.3)	17 (6.7)	5 (5.2)	29 (5.8)	
Agree	7 (21.9)	7 (9.1)	9 (23.1)	55 (21.8)	10 (10.4)	88 (17.7)	
Strongly agree	23 (71.9)	67 (87.0)	24 (61.5)	153 (60.7)	74 (77.1)	341 (68.7)	
One should pay attention to smoking in presence of children							
Strongly disagree	1 (3.1)	4 (5.1)	1 (2.6)	7 (2.8)	4 (4.2)	17 (3.4)	.859
Disagree	0 (0.0)	0 (0.0)	1 (2.6)	3 (1.2)	0 (0.0)	4 (0.8)	
Undecided	0 (0.0)	2 (2.6)	0 (0.0)	3 (1.2)	2 (2.1)	7 (1.4)	
Agree	3 (9.4)	3 (3.9)	1 (2.6)	8 (3.2)	4 (4.2)	19 (3.8)	
Strongly agree	28 (87.5)	69 (88.5)	36 (92.3)	231 (91.7)	86 (89.6)	450 (90.5)	

*chi-square

There is no significant difference regarding whether smoking in front of patients in the work place is ethical, because most of the examinees, 399 (80.3%), consider that this is not ethical. Almost all examinees agree that health care professionals’ ethical responsibility is to warn pregnant women of the harmful effects smoking has on the fetus (Table 5). There was no significant difference regarding the answers to questions wether the health care professionals have a greater responsibility when it comes to prevention of the harmful effects of smoking than other society members (Table 6).

**Table 5:** Attitude toward health care professionals’ ethical responsibility to warn smokers and pregnant women who smoke about harmful effects of smoking

	Resident physicians	Specialist physicians	Trainee nurses	Nurses with secondary education	Nurses with associate degree	Total	p*
	N (%)	N (%)	N (%)	N (%)	N (%)	N (%)	
Health care professionals’ ethical responsibility is to warn the smokers of the harmful effects of smoking							
Strongly agree	1 (3.1)	1 (3.1)	3 (7.9)	11 (4.4)	2 (2.1)	18 (3.6)	.044
Agree	0 (0.0)	2 (2.6)	0 (0.0)	5 (2.0)	1 (1.0)	8 (1.6)	
Undecided	0 (0.0)	2 (2.6)	8 (21.1)	27 (10.8)	9 (9.3)	46 (9.3)	
Agree	8 (25.0)	13 (16.7)	7 (18.4)	62 (24.7)	18 (18.6)	108 (21.8)	
Strongly agree	23 (71.9)	60 (76.9)	20 (52.6)	146 (58.2)	67 (69.1)	316 (63.7)	
Health care professionals’ ethical responsibility is to warn pregnant women of the harmful effects smoking has on the fetus							
Strongly agree	0 (0.0)	1 (3.1)	0 (0.0)	8 (3.2)	2 (2.1)	11 (2.2)	.334
Agree	1 (3.1)	1 (3.1)	0 (0.0)	4 (1.6)	2 (2.1)	8 (1.6)	
Undecided	0 (0.0)	2 (2.6)	0 (0.0)	8 (3.2)	4 (4.2)	14 (2.8)	
Agree	5 (15.6)	5 (6.4)	6 (15.4)	49 (19.4)	10 (10.4)	75 (15.1)	
Strongly agree	26 (81.3)	69 (88.5)	33 (84.6)	183 (72.6)	78 (81.3)	389 (78.3)	
Total	32 (100)	78 (100)	39 (100)	251 (100)	97 (100)	497 (100)	

*chi-square

**Table 6:** Attitudes toward example and responsibility of health care professionals

	Resident physicians	Specialist physicians	Trainee nurses	Nurses with secondary education	Nurses with associate degree	Total	p*
	N (%)	N (%)	N (%)	N (%)	N (%)	N (%)	
Health care professionals are example to their patients							
Strongly disagree	2 (6.2)	5 (6.5)	2 (5.1)	24 (9.6)	4 (4.1)	37 (7.5)	.435
Disagree	2 (6.2)	2 (2.6)	1 (2.6)	10 (3.9)	2 (2.1)	17 (3.4)	
Undecided	4 (12.5)	5 (6.5)	6 (15.4)	41 (16.3)	15 (15.5)	71 (14.3)	
Agree	14 (43.7)	24 (31.2)	11 (28.2)	74 (29.5)	28 (28.8)	151 (30.4)	
Strongly agree	10 (31.2)	41 (53.2)	19 (48.7)	102 (40.7)	48 (49.5)	220 (44.3)	
Health care professionals are example to the society							
Strongly disagree	2 (6.2)	4 (5.2)	2 (5.1)	32 (12.7)	5 (5.1)	45 (9.0)	.088
Disagree	2 (6.2)	2 (2.6)	2 (5.1)	16 (6.3)	2 (2.1)	24 (4.8)	
Undecided	5 (15.6)	9 (11.7)	13 (33.3)	45 (17.9)	18 (18.6)	90 (18.1)	
Agree	11 (34.4)	26 (33.8)	8 (20.5)	70 (27.8)	26 (26.8)	141 (28.4)	
Strongly agree	12 (37.5)	36 (46.7)	14 (35.9)	89 (35.3)	46 (47.4)	197 (39.6)	
Health care professionals have a greater responsibility when it comes to prevention of the harmful effects of smoking than the other society members							
Strongly disagree	1 (3.1)	4 (5.2)	4 (10.3)	25 (10.0)	5 (5.1)	39 (7.9)	.074
Disagree	5 (15.6)	4 (5.2)	1 (2.6)	21 (8.4)	6 (6.2)	37 (7.5)	
Undecided	2 (6.2)	3 (3.9)	10 (25.6)	34 (13.6)	13 (13.4)	62 (12.5)	
Agree	10 (31.2)	19 (24.7)	11 (28.2)	65 (26.1)	22 (22.7)	127 (25.7)	
Strongly agree	14 (43.7)	47 (61.0)	13 (33.3)	104 (41.8)	51 (52.6)	229 (46.4)	
Total	32 (100)	78 (100)	39 (100)	251 (100)	97 (100)	497 (100)	

*chi-square

The influence of gender, age, and workplace on the factors “attitudes toward smoking” and “opinion about the role of healthcare provider in smoking” was tested through multivariate analysis of variance. We have three nominal variables and two continuous variables. Using multivariate analysis we found that there was no statistically significant influence of sex (Wilksov Λ = 0.99, F (2439) = 0.65, *p* > 0.05, η2 = 0.003) or age (Wilksov Λ = 0.97, F (8.880) = 1.53, *p* > 0.05, η2 = 0.014) on the attitude toward smoking and the opinion about the role of healthcare professionals in smoking. On the other hand, there was a statistically significant effect of the workplace on the attitude toward smoking and the opinion about the role of healthcare workers in smoking (Wilksov Λ = 0.93, F (10.880) = 3.86, *p* < 0.01, η2 = 0.034). For better interpretation of results, one-way analysis of variance was performed with Bonferroni correction (*p* < 0.025), separately for each dependent variable, because the variable “workplace” had more than two factor levels. There was a statistically significant difference in the attitude toward smoking with regard to the level of workplace (F (5.477) = 9.76, *p* < 0.01, η2 = 0.076). The difference was between physician specialists (M = 27.23, sd = 3.43), who have a high opinion that smoking is harmful, and nurses/technicians with the secondary level of education (M = 23.64, SD = 5.36). Furthermore, there was a statistically significant difference in the level of workplaces and the opinion about the role of healthcare workers in smoking (F (5.485) = 3.27, *p* < 0.01, η2 = 0.026). The Bonferroni correction has confirmed that physician specialist (M = 12.64, SD = 3.06) averagely agree more with the claim that healthcare professionals are role models to the wider population, unlike nurses/technicians with secondary level of education (M = 11.34, SD = 3.55).

## Discussion

Although the facts about the harmful effect of smoking are well-known, and health care providers are the first expected to be an example of awareness and consciousness, the data of this research shows a high prevalence of smokers among health care professionals, results similar to previous studies in Croatia [28, 31]. The higher smoking prevalence among health care professionals than that of the general population can potentially be explained by occupational stress, which is one of the key factors to addiction [32].

Occupational stress is mostly related to the nurses who finished secondary school, nursing shortage, nurses who have a large number of overtime working hours, also, nurses with the most shift working hours and the lowest level of education.

One of the key factors is the influence of cultural norms because not so long ago smoking was socially acceptable and highlighted as a specific status symbol.

Health care professionals are familiar with the harmful effects of smoking and passive smoking. The fact that only one of our examinees smokes in front of patients is very encouraging although it is not consistent with other studies where it was found that there are more health care professionals who smoke in front of patients [33]. Out of the total number of participants, 450 (90.5%) of them completely agrees on the issue that smokers should pay attention to smoking in the presence of children. Almost all of the examinees agree with the claim that the medical health professionals have an ethical responsibility to warn pregnant women who smoke of the potentially harmful effects smoking can have on the fetus during pregnancy. Those are results similar to other studies where physicians showed willingness for a conversation with a pregnant woman about adverse effects of smoking and advising about cessation [34].

The great number of physicians completely agree that healthcare professionals have an ethical responsibility to warn smokers of the harmful effects of smoking. However, health care professionals who smoke feel discomfort in situations when they are supposed to counsel their patients about smoking cessation and the harmfulness of the tobacco smoke [35]. They have difficulties in creating transfer/countertransference relationship with patients in the smoking prevention [36]. Problems can also be caused by the lack of motivation of the health care professionals due to previous negative experiences when patients didn’t accept the advice given by them regarding the use of tobacco products for cessation. On the other hand, health care professionals who have been additionally educated and acquainted with problems of smoking cessation, who understand their patients’ problems, and have the will and desire to encourage them to stop smoking, personally feel more content [37].

Physicians and nurses, as a part of the health care system, play an equally important role in the prevention and education of patients about the harmfulness of smoking. Primary care physicians play a major role in the process of making the decision to stop smoking and should be the first one whom the patient turns to. The importance and responsibility of the visiting nurses are the key to promoting the healthy way of life and healthy behaviour. Their role in the community is significant because they participate in giving advice, encouraging people, supporting people and referring them to services which can help them with smoking cessation and pharmacological treatment [38]. Nurses that have been educated had more expertise and better results in interventions for smoking cessation. The studies show that the lack of time and education are the two main reasons that are preventing nurses in giving advice regarding the smoking cessation [39]. Nurses in our study showed disagreement with claims that passive smoking is harmful to health and that one should pay attention to smoking in the presence of non-smokers. Those findings confirm that inadequate knowledge about smoking and smoking cessation affect nurses’ health promotion role. The main reasons for the high prevalence of smoking among nurses include occupational stress, nurse education, professional socialization and lack of support [40]. Although nurses fulfil important roles in health promotion, their potential is under-utilized due to poor knowledge, lack of confidence and a conflict of personal attitudes to smoking [41].

To improve the existing anti-tobacco strategies, the recommendations are to strengthen legislation for tobacco control in hospitals, and to expand current control efforts based on evidence of high-quality data, creating a smoke-free environment where social acceptability of smoking would be reduced. Also, regular surveillance of those programmes along with the dissemination of research findings would provide policy makers evidence that suggests the need for increasing tobacco control achievements [42].

Health care professionals who smoke have difficulty with insufficient education and insufficient motivation due to poor prior experiences when their patients have not accepted their advice on cessation of tobacco product consumption. In our society we need to raise the awareness of citizens and health workers about smoking hazards, so that every person can act responsibly. Health care professionals who are more informed and educated, are familiar with the problem of smoking cessation, understand the patient’s problems, have a stronger will and desire to encourage smoking cessation.

## Conclusions

The results of our research have shown that the prevalence of smokers among health care professionals is high, implying the ineffectiveness of the current anti-tobacco strategy. Health care professionals, who should inform others about the harmfulness of the tobacco smoke, are not fully aware to what extent smoking is harmful both to them and to people who surround them. It would be helpful to implement training programs for healthcare workers to improve their ability in smoking cessation counselling techniques to provide active support to their patients.
